# Supplementary material for: Decoupling of brain function from structure reveals regional behavioral specialization in humans
Source: Nat Commun. 2019 Oct 18;10:4747. doi: 10.1038/s41467-019-12765-7 (PMC6800438; doi:10.1038/s41467-019-12765-7)
Supplement: Supplementary file 1 — Supplementary Information [file 41467_2019_12765_MOESM1_ESM.pdf]

Decoupling of brain function from structure reveals regional behavioral specialization in humans

Maria Giulia Preti & Dimitri Van De Ville

Supplementary Information

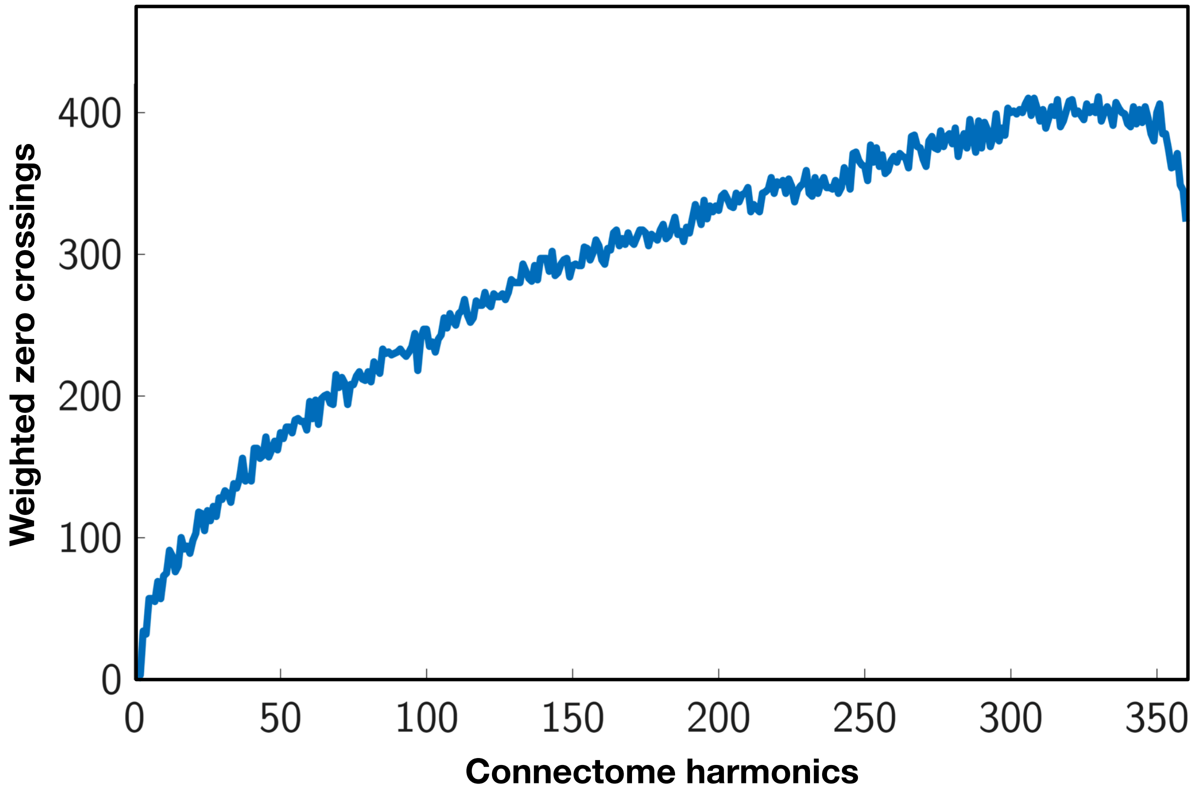

Supplementary Figure 1: Weighted zero crossings along graph structure for each structural harmonic. The increasing complexity of harmonic patterns with increasing index goes along with the notion of higher spatial frequency, and is quantified by the increased number of zero crossings within the pattern. Source data to reproduce this figure are provided as a Source Data file.

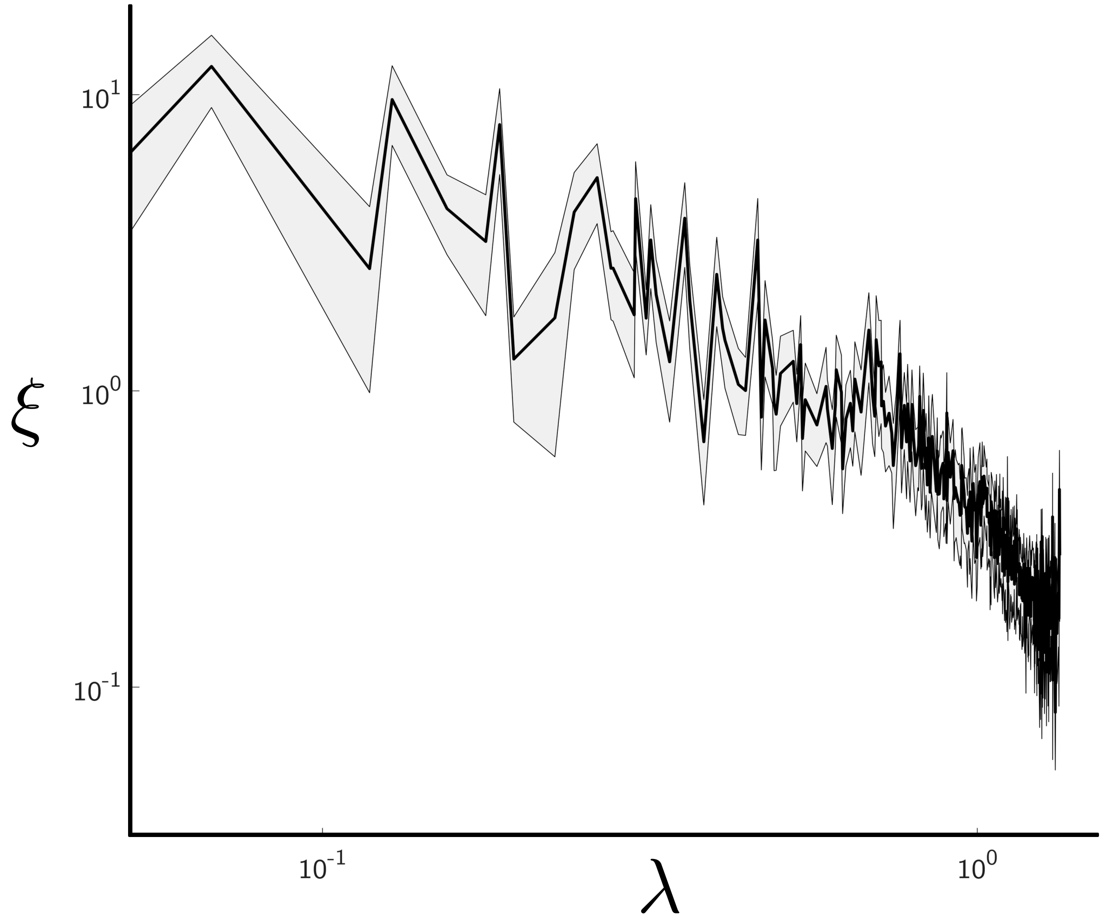

Supplementary Figure 2: Average energy spectral density of resting-state functional data projected on the structural harmonics. Since the density is shown on log-log axes, the two linear trends are reminiscent of power-law regimes. The black line and gray intervals display the mean value $\pm$ standard deviation across subjects. λ = harmonic frequency, ξ = energy. Source data to reproduce this figure are provided as a Source Data file.

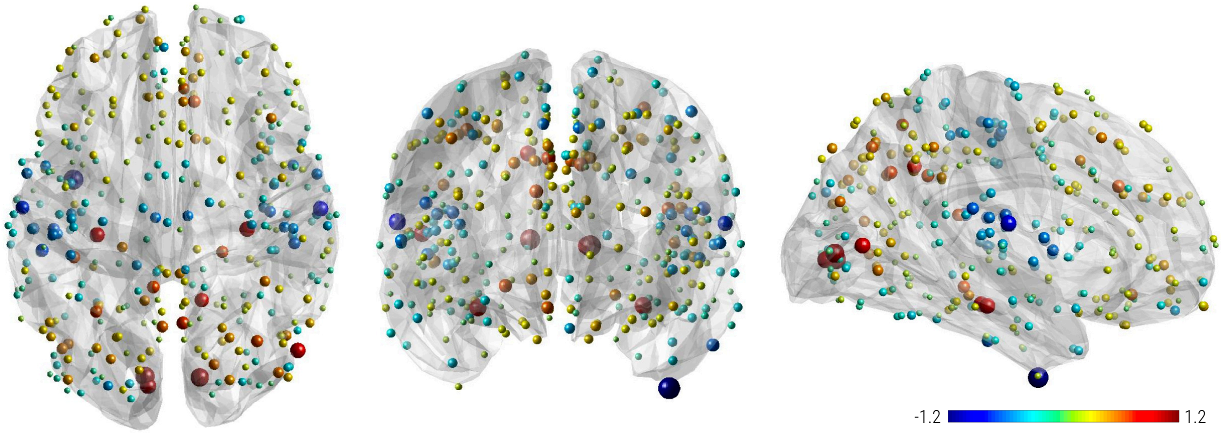

Supplementary Figure 3: Difference between empirical and surrogate structural coupling. The plot shows the binary logarithm of the ratio between the structural-decoupling index computed from empirical activity (shown in Fig. 2c) and from SC-informed surrogates (shown in Fig. 2b). It is noticeable that some of the visual areas (which appear here in red) detach from the rest of the sensory-motor network (shown in blue). Source data to reproduce this figure are provided as a Source Data file.

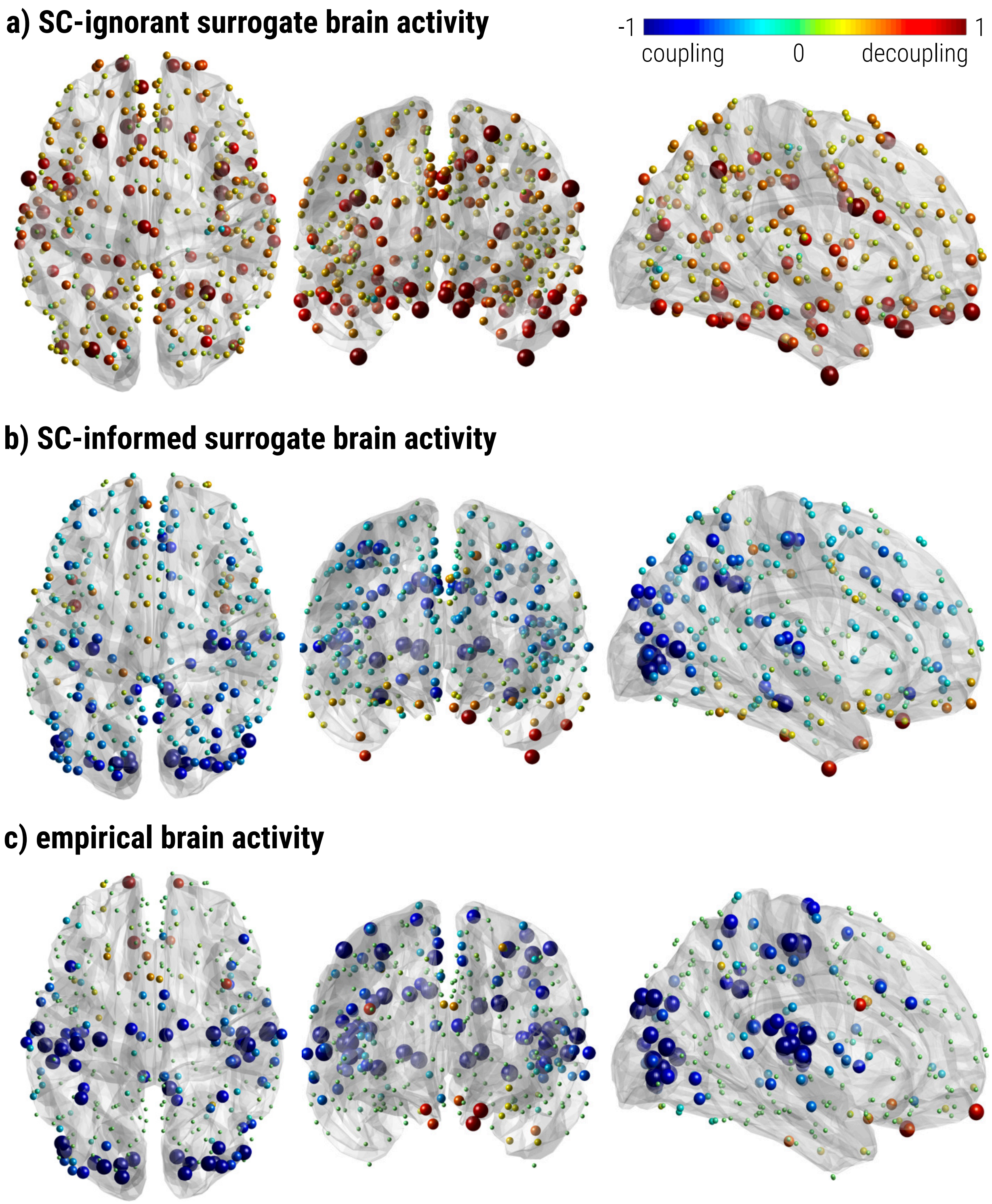

Supplementary Figure 4: Structural-decoupling index computed from the second resting-state

dataset. As for Fig. 2, the binary logarithm of the structural-decoupling index is plotted here for: a) surrogate brain activity timecourses without knowledge of the empirical structural connectome; b) surrogate brain activity timecourses with knowledge of the underlying structural connectome, build as a linear combination of structural harmonics with randomized coefficient signs; c) empirical brain activity. Results are highly reliable across the two test-retest sessions (spatial correlation = 0.99 for (a) and (b), 0.90 for (c)). Source data to reproduce this figure are provided as a Source Data file.

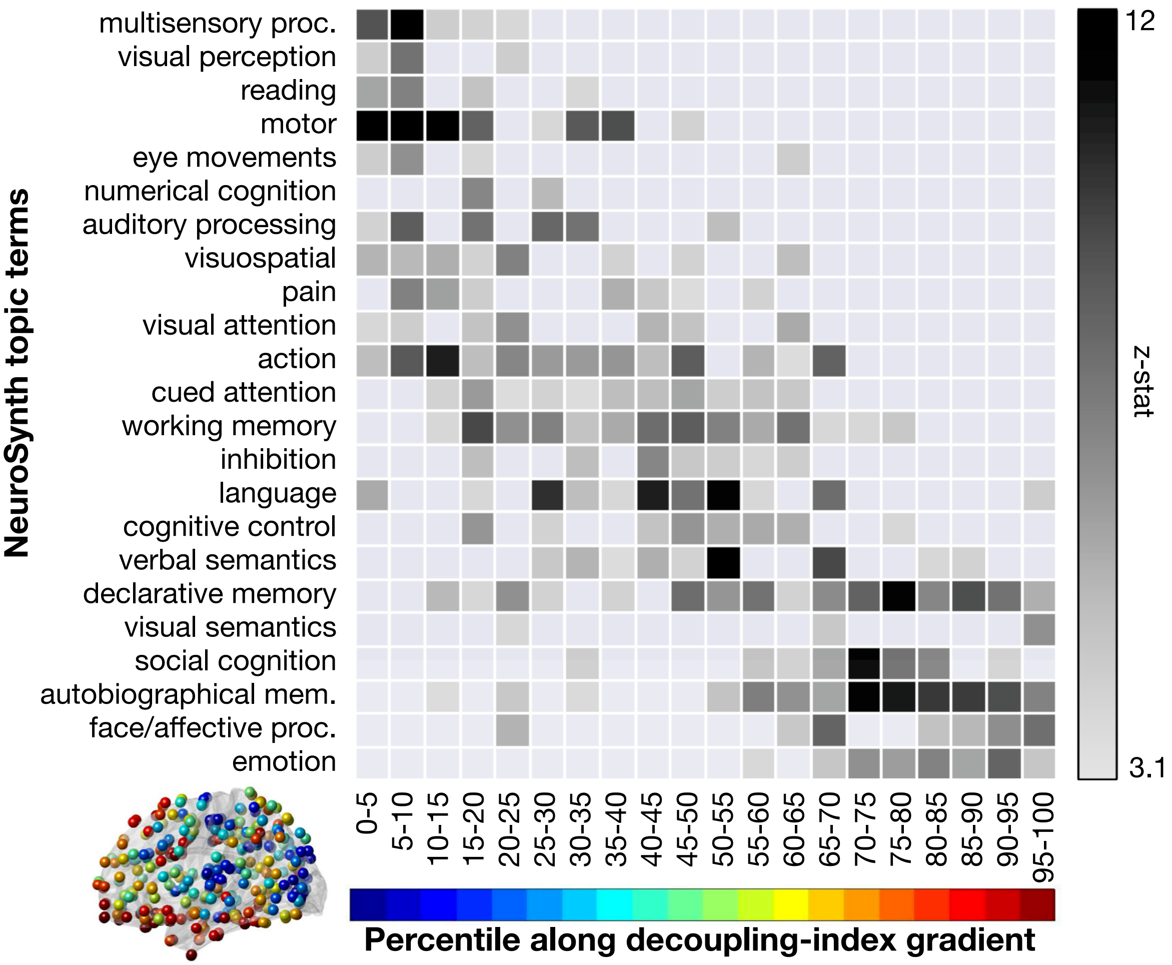

Supplementary Figure 5: NeuroSynth meta-analysis applied to the structural-decoupling index gradient computed from the second dataset. Findings are really highly consistent with the ones of the first dataset, reported in Fig. 3a. Source data to reproduce this figure are provided as a Source Data file.
