# Supplementary material for: Decoupling of brain function from structure reveals regional behavioral specialization in humans
Source: Nat Commun. 2019 Oct 18;10:4747. doi: 10.1038/s41467-019-12765-7 (PMC6800438; doi:10.1038/s41467-019-12765-7)
Supplement: Supplementary file 3 — Reporting Summary [file 41467_2019_12765_MOESM3_ESM.pdf]

nature research

Corresponding author(s): Maria Giulia Preti

Last updated by author(s): Aug 26, 2019

Reporting Summary

Nature Research wishes to improve the reproducibility of the work that we publish. This form provides structure for consistency and transparency in reporting. For further information on Nature Research policies, see Authors & Referees and the Editorial Policy Checklist .

Statistics

For all statistical analyses, confirm that the following items are present in the figure legend, table legend, main text, or Methods section.

n/a	Confirmed	
☐	☒	The exact sample size (*n*) for each experimental group/condition, given as a discrete number and unit of measurement
☐	☒	A statement on whether measurements were taken from distinct samples or whether the same sample was measured repeatedly
☐	☒	The statistical test(s) used AND whether they are one- or two-sided<br>*Only common tests should be described solely by name; describe more complex techniques in the Methods section.*
☐	☒	A description of all covariates tested
☐	☒	A description of any assumptions or corrections, such as tests of normality and adjustment for multiple comparisons
☐	☒	A full description of the statistical parameters including central tendency (e.g. means) or other basic estimates (e.g. regression coefficient) AND variation (e.g. standard deviation) or associated estimates of uncertainty (e.g. confidence intervals)
☐	☒	For null hypothesis testing, the test statistic (e.g. *F*, *t*, *r*) with confidence intervals, effect sizes, degrees of freedom and *P* value noted<br>*Give P values as exact values whenever suitable.*
☒	☐	For Bayesian analysis, information on the choice of priors and Markov chain Monte Carlo settings
☒	☐	For hierarchical and complex designs, identification of the appropriate level for tests and full reporting of outcomes
☐	☒	Estimates of effect sizes (e.g. Cohen's *d*, Pearson's *r*), indicating how they were calculated

Our web collection on statistics for biologists contains articles on many of the points above.

Software and code

Policy information about availability of computer code

Data collection	No software was used to acquire the data. The data is supplied by the Human Connectome Project (HCP) publicly available database (https://db.humanconnectome.org).
Data analysis	Custom-code was implemented in Matlab R2016b and Python 2.7.15. The full code necessary to reproduce all results and paper figures is available here: https://github.com/gpreti/GSP_StructuralDecouplingIndex.

For manuscripts utilizing custom algorithms or software that are central to the research but not yet described in published literature, software must be made available to editors/reviewers. We strongly encourage code deposition in a community repository (e.g. GitHub). See the Nature Research guidelines for submitting code & software for further information.

Data

Policy information about availability of data

All manuscripts must include a data availability statement. This statement should provide the following information, where applicable:

- Accession codes, unique identifiers, or web links for publicly available datasets
- A list of figures that have associated raw data
- A description of any restrictions on data availability

The MRI data that support the findings of this study can be downloaded from the Human Connectome Project (HCP) publicly available database (https://db.humanconnectome.org). The ID of the subjects are reported here: https://github.com/gpreti/GSP_StructuralDecouplingIndex.

Field-specific reporting

Please select the one below that is the best fit for your research. If you are not sure, read the appropriate sections before making your selection.

Life sciences study design

All studies must disclose on these points even when the disclosure is negative.

Sample size	220 acquisitions from the HCP (56 subjects and 4 fMRI sessions each, 4 excluded for incomplete acquisition) represent a large sample for fMRI studies. The aim is to report consistent results across subjects. No subject-specific features or traits are used.
Data exclusions	No data were excluded from the analysis.
Replication	The results were replicable across different samples (smaller preliminary sample of 21 acquisitions, then bigger ones of 56 and 112). A test-retest analysis between different sessions should very high reliability (results included in the manuscript).
Randomization	We do not use subject randomization to allocate participants in different experimental groups, as we only have one experimental group.
Blinding	There was no group allocation of individuals, as we only have one experimental group.

Reporting for specific materials, systems and methods

We require information from authors about some types of materials, experimental systems and methods used in many studies. Here, indicate whether each material, system or method listed is relevant to your study. If you are not sure if a list item applies to your research, read the appropriate section before selecting a response.

Materials & experimental systems

n/a	Involved in the study	
☒	☐	Antibodies
☒	☐	Eukaryotic cell lines
☒	☐	Palaeontology
☒	☐	Animals and other organisms
☐	☒	Human research participants
☒	☐	Clinical data

Methods

n/a	Involved in the study	
☒	☐	ChIP-seq
☒	☐	Flow cytometry
☐	☒	MRI-based neuroimaging

Human research participants

Policy information about studies involving human research participants

Population characteristics	Age range 26-35, M/F 22/34.
Recruitment	Subjects were selected from the HCP database with the following criteria: age between 26 and 35 years old, cognitive status (MMSE) > 28, image reconstruction info = 3T MR r227.
Ethics oversight	All experiments were reviewed and approved by the local institutional ethical committee (Swiss Ethics Committee on research involving humans). Informed consent forms, including consent to share de-identified data, were collected for all subjects (within the HCP) and approved by the Washington University institutional review board. All methods were carried out in accordance with relevant guidelines and regulations.

Note that full information on the approval of the study protocol must also be provided in the manuscript.

Magnetic resonance imaging

Experimental design

Design type	Resting-state.
Design specifications	1200 frames acquired per resting-state session, four sessions of 14.4 minutes included.
Behavioral performance measures	No behavioral performance is assessed, as we do not have a task paradigm.

Acquisition

Imaging type(s)	Functional, structural, diffusion MRI.
Field strength	3T
Sequence & imaging parameters	Structural 3D MPRAGE T1-weighted sequence:TR=2400ms, TE=2.14ms, TI=1000, flip angle=8deg, FOV=224x224mm, voxel size=0.7mm isotropic. Resting-state gradient-echo EPI sequences: 1200 frames, TR=720ms, TE=3.31ms, flip angle=52deg, FOV=208x180mm, voxel size=2mm isotropic, multiband factor=8. Diffusion weighted sequence: spin-echo EPI, TR=5520ms, TE=89.5ms, flip angle=78◦, FOV=208x180.
Area of acquisition	Whole-Brain.

Diffusion MRI ☒ Used ☐ Not used

Parameters: 3 shells of b=1000, 2000, 3000 s/mm2 with 90 directions plus 6 b=0 acquisitions.

Preprocessing

Preprocessing software	Matlab R2016b and toolboxes SPM8, DPARSF.
Normalization	We started from the minimally preprocessed datasets supplied from HCP, in which spatial distortions have been minimized and the data have been aligned across modalities and across subjects to Montreal Neurological Institute (MNI) standard space using appropriate volume-based and surface-based registration methods (see HCP documentation for details).
Normalization template	Montreal Neurological Institute (MNI).
Noise and artifact removal	Voxelwise timecourses were detrended (linear and quadratic trends) and nuisance variables were regressed out using the DPARSF toolbox. These included six motion parameters and average white matter and cerebrospinal fluid signals, obtained from standard white matter and ventricular masks mapped to the subjects' fMRI space and masked with individual segmentation maps.
Volume censoring	No censoring.

Statistical modeling & inference

Model type and settings	General linear model performed only for nuisance regression (see noise and artifact removal). Voxelwise timecourses are considered then for atlas-based functional connectivity analysis. No inference/effect tested.
Effect(s) tested	None, non relevant.

Specify type of analysis: ☐ Whole brain ☒ ROI-based ☐ Both

Anatomical location(s): Glasser's multi-modal cortical atlas (Glasser et al., Nature, 2016) converted to volume was used to parcellate the cortex into N = 360 ROIs and generate the functional and structural connectomes.

Statistic type for inference (See Eklund et al. 2016)	None, non relevant.
Correction	None, non relevant.

Models & analysis

n/a	Involved in the study
☐	☒ Functional and/or effective connectivity
☐	☒ Graph analysis
☒	☐ Multivariate modeling or predictive analysis

Functional and/or effective connectivity	Pearson correlation was computed between regionally-averaged timecourses (360 ROIs of Glasser parcellation).
Graph analysis	From the diffusion acquisitions, weighted structural connectivity graphs were build. The chosen connectivity measure was the number of fibers connecting two regions divided by the region volumes (sum of connected regions). A group connectome was obtained by averaging all subjects' structural matrices.
